# In morphea, cytotoxic resident memory T cells induce chronic, immunogenic endothelial cell injury via necroptosis

**DOI:** 10.1172/jci.insight.201949

**Published:** 2026-04-02

**Authors:** William J. Crisler, Noor Sohail, Samuel J. Steuart, Maria Vazquez-Machado, Arjun Mahajan, Maureen Whittelsey, Alex Pickering, Michael J. Martinez, Theresa Hutchins, Jessica E. Teague, Qian Zhan, Shannan Ho Sui, Ruth Ann Vleugels, Kathryn S. Torok, Heidi Jacobe, Rachael A. Clark, Avery LaChance

**Affiliations:** 1 Brigham and Women’s Hospital and Harvard Medical School, Boston, Massachusetts, USA.; 2 Harvard Chan School of Public Health, Boston, Massachusetts, USA.; 3 University of Pittsburgh Medical Center, Pittsburgh, Pennsylvania, USA.; 4 University of Texas Southwestern Medical Center, Dallas, Texas, USA.

**Keywords:** Dermatology, Immunology, Cell stress, Cellular immune response, Fibrosis

## Abstract

Chronic skin fibrosis in morphea results from resident memory T cells that kill endothelial cells through inflammatory cell death and sustain injury and scarring.

**To the Editor:** Morphea is a skin-limited fibrotic disorder histologically identical to systemic sclerosis (SSc). Its mechanisms are poorly defined. Chronic endothelial injury appears to play a central role in both initiating and sustaining fibrosis in SSc (1). We recently reported that established, fibrotic skin lesions of morphea exhibit ongoing antigen-driven cytotoxic T cell injury despite similar T cell numbers compared with healthy skin, suggesting a small population drives injury (2). Tissue-resident memory T cells (Trm) are long-lived, nonrecirculating lymphocytes that can exhibit outsized effects even in small numbers. The roles of Trm and endothelial injury in morphea fibrosis remain unclear.

To define T cell subsets in morphea, we analyzed published single-cell RNA-seq (scRNA-seq) data from 13 morphea and 8 healthy skin (HS) biopsies (Figure 1A, Supplemental Figure 1A, and Supplemental Table 1; supplemental material available online with this article; https://doi.org/10.1172/jci.insight.201949DS1) (3). T cell numbers and frequencies were similar between groups (Figure 1B). Clustering defined CD8^+^ and CD4^+^ Trm subsets expressing *CD69* and *LGALS3* (Figure 1C and Supplemental Figure 1B). Cytotoxic CD8^+^ Trm in morphea expressed *GZMB*, *GZMA*, *NKG7*, and *IFNG*, consistent with a terminal effector phenotype (Figure 1D).

We next examined endothelial cells (ECs) in the scRNA-Seq dataset (Figure 1E and Supplemental Figure 1, C and D). Excluding lymphatic ECs and pericytes, pooled vascular ECs (VECs) in morphea displayed a coordinated stress program. Differential expression showed increased mitochondrial/redox stress (*AKR1C1*, *MAOB*), integrated stress response (*RPL26*, *MRPS17*), nucleolar stress (*NOL12*), and autophagy (*GABARAP*) genes, with reduced endothelial stability and repair signals (*NRARP*, *BMP2*, *AKAP12*) (Figure 1F). *TNFRSF1A* increased and *TNFAIP3* decreased, indicating susceptibility to TNF-mediated signaling (Figure 1F). Ingenuity Pathway Analysis predicted activation of oxidative phosphorylation, respiratory electron transport, the integrated stress response, and mitochondrial protein degradation (Supplemental Figure 1E). Upstream regulator analysis indicated AMPK-axis metabolic stress with suppression of mTORC1-driven translation and endoplasmic reticulum-associated ubiquitin-fold modifier 1 conjugation (ER UFMylation), consistent with disrupted protein homeostasis under stress (Supplemental Figure 1F). These data indicate that ECs in morphea are metabolically strained, TNF sensitized, and have diminished homeostatic repair capacity, a pattern that mirrors endothelial injury responses described under cytokine or oxidant stress (4).

Morphea lesions exhibit enriched TNF pathway activity (2). Predicted ligand-receptor interactions in morphea implicated T cells as sources of TNF and ECs as recipients via TNFRSF1A and TNFRSF1B (Figure 1G and Supplemental Figure 1G). TNF signaling can trigger inflammatory cell death pathways, including necroptosis (5). Consistent with immune-mediated endothelial injury, scRNA-seq revealed loss of postcapillary venule ECs and a relative increase of other venular ECs (Figure 1H and Supplemental Figure 1H).

Given the cytotoxic T cell signature and evidence of endothelial stress, we performed multiplex immunostaining to determine if ECs were undergoing T cell–associated death in morphea lesions (Supplemental Table 2). Endothelial apoptosis was increased in morphea lesions, with increased counts and percentages of CD31^+^cleaved-caspase-3^+^ and CD31^+^cleaved-PARP^+^ cells and more T cells adjacent to apoptotic ECs (Figure 1I and Supplemental Figure 2, A–C). We previously identified a strong necroptosis transcriptional signature in morphea (1). Immunostaining revealed increased numbers of active necroptotic (pMLKL^+^) ECs (CD31^+^) near cytotoxic (CD8^+^) and tissue-resident (CD69^+^ or CD103^+^) T cells (CD3^+^) (Figure 1, J and K). Trm numbers did not differ between groups, indicating heightened cytotoxic activity rather than Trm expansion, and endothelial necroptosis was increased after normalization to total CD31^+^ cells (Supplemental Figure 2, D–H). Further supporting endothelial necroptosis in morphea, CD31^+^ ECs adjacent to T cells coexpressed pMLKL and RIPK3 (Supplemental Figure 2I).

In summary, we find evidence of ongoing Trm-mediated, immunogenic EC death in the pauci-inflammatory, fibrotic skin lesions of morphea. We propose that T cells recruited into the skin during the earlier inflammatory phases of morphea may persist as cytotoxic Trm in established fibrotic lesions, possibly mediating chronic, immunogenic EC death and perpetuating fibrosis (Supplemental Figure 2J). Inhibitors of necroptosis appear to be well tolerated and are now in clinical trials for the treatment of multiple inflammatory diseases, including psoriasis, rheumatoid arthritis, and ulcerative colitis (6). Our studies suggest that inhibition of necroptosis or depletion of pathogenic Trm may be novel therapeutic strategies for the treatment of morphea and possibly other fibrotic disorders.

Detailed methods, including information on statistical analyses, study approval, data availability, and acknowledgments, are provided in the Supplemental Methods.

## Funding support

This work is the result of NIH funding and is subject to the NIH Public Access Policy. Through acceptance of this federal funding, the NIH has been given a right to make the work publicly available in PubMed Central.

- NIH National Institute of Arthritis and Musculoskeletal and Skin Diseases P30AR069625 (to RC).
- Dermatology Foundation Research Career Development Award (to WC).
- Dermatology Foundation Medical Dermatology Career Development Award (to AL).

## Conflict of interest

AL has received research funds from Pfizer and Merck, and consulting funds from Johnson & Johnson, Pfizer, MEDACorp, Guidepoint, and TD Cowen.

## Supplementary Material

Supplemental data

Supporting data values

## Figures and Tables

**Figure 1:**
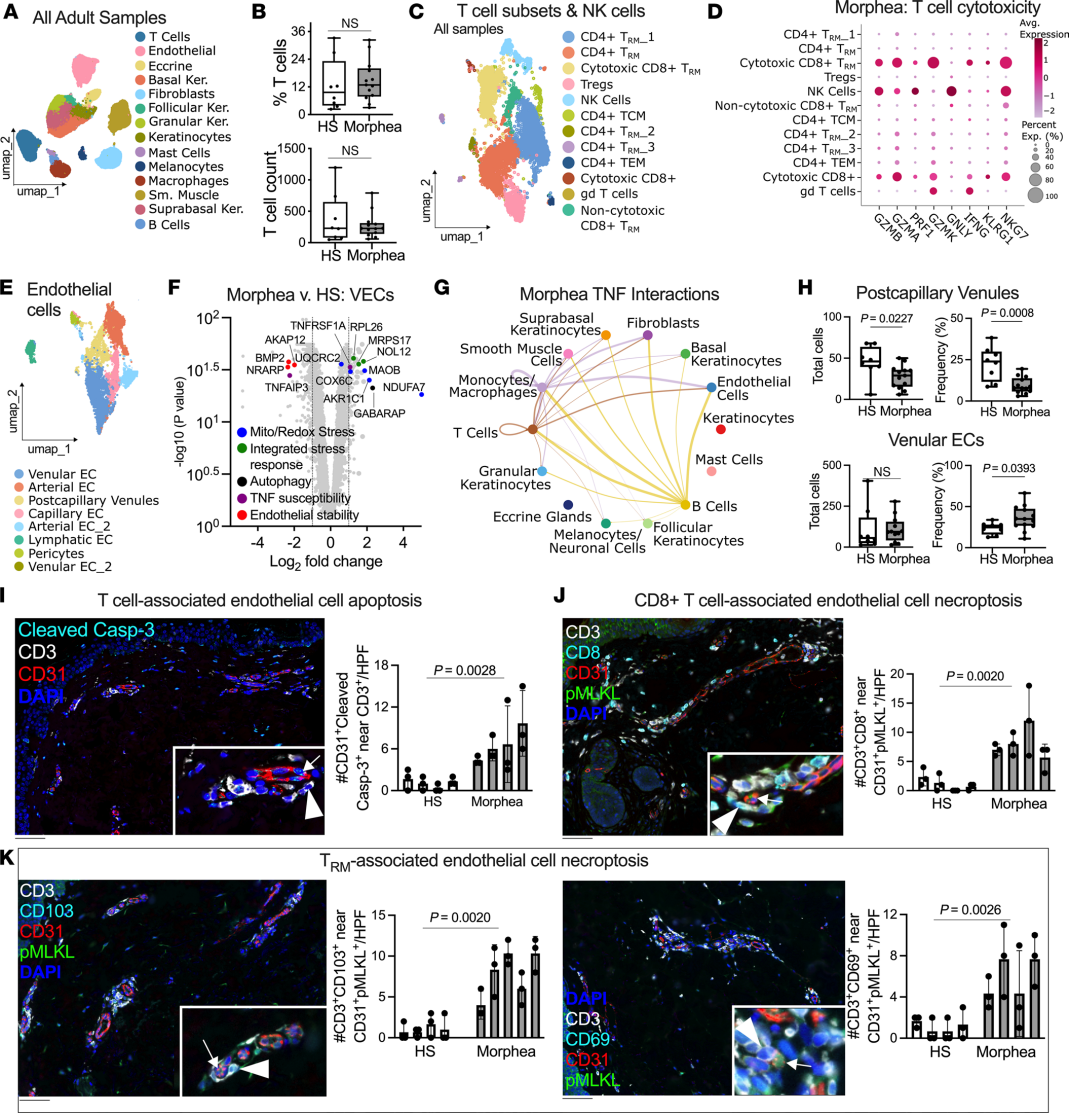
Cytotoxic Trm induce endothelial injury and necroptosis in morphea skin lesions. (**A**–**F**) Single-cell RNA-seq (scRNA-Seq) studies from lesions of adult morphea (*n* = 13) and healthy skin (HS) (*n* = 8) biopsies showing major skin cell populations (**A**), T cell counts and frequencies (**B**), T cell and NK cell subsets (**C**), cytotoxic gene expression across T cell subsets in morphea (**D**), endothelial cell (EC) subsets (**E**), and differentially expressed genes (**F**) in pooled vascular ECs from morphea versus HS. (**G**) CellChat analysis predicted TNF ligand-receptor interactions among all cell populations in morphea, with thicker lines indicating stronger interaction strength; only significant interactions are shown. (**H**) Postcapillary venule ECs are reduced in morphea, with a relative increase of other venular endothelial cells. (**I**) Multiplex immunofluorescence shows apoptotic (cleaved-caspase-3^+^) ECs (CD31^+^) adjacent to CD3^+^ T cells in morphea, with quantification per 200× high-power field (HPF) shown. (**J** and **K**) Representative morphea images show necroptotic (pMLKL^+^) ECs near CD3^+^CD8^+^ cytotoxic T cells (**J**) and CD3^+^CD69^+^ or CD3^+^CD103^+^ tissue-resident T cells (**K**), with corresponding quantification per 200× HPF versus HS. Insets: arrowheads indicate T cells; arrows indicate apoptotic (**I**) or necroptotic (**J** and **K**) ECs. Scale bars: 50 μm. Bars represent individual donors; data are shown as mean ± SEM of 3 independent measurements. Statistical significance for immunostaining was determined by nested 2-tailed *t* tests; for box plots, by 2-tailed unpaired *t* tests.

## Version 1 04/02/2026

In-Press Preview

## Version 2 05/22/2026

Electronic publication
