# Supplementary material for: In morphea, cytotoxic resident memory T cells induce chronic, immunogenic endothelial cell injury via necroptosis
Source: JCI Insight. 2026 Apr 2;11(10):e201949. doi: 10.1172/jci.insight.201949 (PMC13232711; doi:10.1172/jci.insight.201949)
Supplement: Supplemental data [file jciinsight-11-201949-s052.pdf]

Supplemental Material

In morphea, cytotoxic resident memory T cells induce chronic, immunogenic endothelial cell injury via necroptosis

William J. Crisler[1], Noor Sohail[2], Samuel J. Steuart[1], Maria Vazquez-Machado[1], Arjun Mahajan[1], Maureen Whittelsey[1], Shannan Ho Sui[2], Alex Pickering[3], Michael J. Martinez[1], Theresa Hutchins[4], Jessica Teague[1], Qian Zhan[1], Ruth Ann Vleugels[1], Kathryn S Torok[4], Heidi Jacobe[5], Rachael A. Clark[1*], Avery LaChance[1*]

[1] Department of Dermatology, Brigham and Women's Hospital, Harvard Medical School, Boston, MA, USA

[2] Harvard Bioinformatics Core, Department of Biostatistics, Harvard T.H. Chan School of Public Health, Boston, MA, USA.

[3] Department of Biomedical Informatics, Harvard Medical School, Boston, MA, USA

[4] Division of Pediatric Rheumatology, University of Pittsburgh Medical Center, Pittsburgh, PA, USA

[5] Department of Dermatology, University of Texas Southwestern Medical Center, Dallas, TX, USA

*Co-senior authors

Methods

Sex as a Biological Variable

Samples for the single-cell dataset were obtained from both male (n = 9) and female (n = 12) participants. Samples for the immunostaining dataset were also obtained from both male (n = 1) and female (n = 10) participants. Sex was recorded at collection but not analyzed separately, as sex-based differences were not the focus of this study.

Study Design

This study was designed to define the presence of cytotoxic tissue-resident memory T cells (T_{RM}) in morphea skin, to assess endothelial cell injury, and to investigate the relationship between T_{RM} cytotoxicity and endothelial cell necroptosis. Single-cell RNA sequencing (scRNA-seq) data from a published dataset (1) containing pediatric and adult samples were reanalyzed. This study focused exclusively on adult morphea and healthy control skin to define endothelial and T-cell subsets and identify transcriptional signatures of endothelial activation and stress. Formalin-fixed, paraffin-embedded (FFPE) skin biopsies from patients with morphea evaluated at Brigham and Women's Hospital (BWH) and healthy adult donors were immunostained to visualize spatial relationships between T_{RM} and apoptotic or necroptotic endothelial cells.

Patient and Skin Biopsy Details

ScRNA-Seq data were obtained from published datasets of adult morphea and healthy control skin obtained at the University of Texas Southwestern Medical Center (UTSW) and the University of Pittsburgh Medical Center (UPMC) (1). Raw and processed data were shared directly by the study authors and imported into the Cellenics platform. Metadata for all scRNA-

Seq samples, including age, sex, and biopsy site, are summarized in Table 1. Archived FFPE skin biopsies from patients with morphea evaluated at the Brigham and Women's Hospital (BWH) were identified through the Mass General Brigham Research Patient Data Registry and retrieved from the BWH pathology archives for immunostaining studies. Metadata for tissues used in immunostaining are summarized in Table 2.

Single-cell RNA-seq analysis using Cellenics®

Single-cell RNA-seq data from lesional morphea and healthy control skin biopsies were analyzed using the Cellenics® platform, hosted and developed by Harvard Medical School (https://github.com/hms-dbmi-cellenics). Analyses were performed between May 2025 and October 2025. The Cellenics pipeline has been described previously (2). Briefly, Cellenics implements a Seurat-based workflow that includes quality control, normalization, batch correction with Harmony, dimensional reduction, and clustering. For this study, prefiltered count matrices were uploaded to Cellenics for visualization and differential-expression analyses. Prior to upload, subsetting of T cell and endothelial populations, Harmony integration, and re-clustering using the Louvain algorithm were performed independently in R with Seurat. The resulting processed objects were then used to generate UMAPs, dot plots, and differential-expression results within Cellenics. The adult dataset included 6,550 T cells (3,559 from morphea and 2,991 from healthy skin) and 7,809 endothelial cells (4,448 from morphea and 3,361 from healthy skin).

Cell type annotation was performed manually based on canonical marker gene expression. For T cell subsets, *CD3D* and *TRAC* identified T cells; *FOXP3* defined regulatory T cells (Tregs);

CD69 and *LGALS3* (3) identified T_{RM}; *SELL* and *CCR7* defined central memory T cells (TCM); *GZMB*, *PRF1*, and *GNLY* marked cytotoxic populations; and *TRDC* identified γδ T cells. Hutchins et al. (4) analyzed pooled adult and pediatric morphea samples from these same datasets to characterize endothelial populations. We focused on the adult samples to define endothelial stress programs and relate these states to spatial evidence of injury. For endothelial cell (EC) subsets, canonical endothelial markers (*PECAM1*, *VWF*, *CLDN5*) defined the EC compartment. Identified clusters included venular ECs (*CLDN5*, *KLF2*, *ESAM* with low *ACKR1*, *SELE*, *ICAM1*, *NFKBIA*, and *IL6*), arterial ECs (*EFNB2* and *SOX17*), postcapillary venules (*ACKR1*, *SELE*, *ICAM1*, *NFKBIA*, and *IL6*), lymphatic ECs (*LYVE1*), and pericytes (*ACTA2*). Differential expression and IPA used pooled vascular ECs after excluding lymphatic ECs and pericytes.

CellChat

CellChat (v2.2.0) (5) was used to infer intercellular signaling within the 13 morphea skin samples analyzed by single-cell RNA sequencing. The Seurat object was converted into a CellChat project, and the human ligand-receptor database was applied to identify significant interactions among cell clusters. Default parameters were used except during the *computeCommunProb* step, where a truncated mean approach (type = "truncatedMean") was applied with a contact range of 100 and no distance scaling (distance.use = FALSE). Analyses were restricted to morphea samples to define intra-lesional signaling networks. TNF-related interactions were visualized using CellChat's chord and dot plot functions. Predicted TNF signaling interactions were considered significant if the CellChat permutation test yielded $P < 0.05$.

Immunostaining

Phosphorylation of MLKL is required for necroptosis activation and can only be assessed at the protein level. ScRNA-Seq captures predominantly viable cells and lacks information on protein post-translational modifications, so immunostaining was used to evaluate dying and necroptotic endothelial cells in tissue. Immunostaining was performed as previously described (6). All samples within each staining panel were processed and imaged in parallel. FFPE sections were stained with antibodies against CD3 (mouse IgG2b, clone C3e/1308, Novus NBP2-53387, 1:100), CD31 (mouse IgG1, clone 89C2, Cell Signaling 3528, 1:800; or rabbit IgG, Abcam ab28364, 1:200), CD8 (mouse IgG1, clone C8/144B, Dako M7103, 1:100), CD69 (mouse IgG1, clone 8B6, Novus NBP1-51607, 1:100), CD103 (rabbit IgG, Abcam ab129202, 1:500), granzyme B (mouse IgG2a, Invitrogen, 1:100), cleaved caspase-3 (rabbit IgG, Cell Signaling 9579, 1:100), cleaved PARP (mouse IgG1, clone E2T4K, Cell Signaling 32563S, 1:50), RIPK3 (rabbit IgG, clone E7A7F, Cell Signaling 10188, 1:100), and phosphorylated MLKL (mouse IgG2a, clone 954702, R&D MAB9187, 1:100). Images were captured on a Mantra Quantitative Pathology Workstation (PerkinElmer) with Mantra Snap 1.0, then processed in InForm 2.4.8 (Akoya Biosciences). To minimize batch effects, all images within a given panel were acquired in a single session with identical microscope settings (laser intensity, exposure time, gain, and filter sets). Spectral unmixing in InForm reduced autofluorescence and background before quantification.

Cells of interest were quantified in 200× high-power fields (HPF). Each sample had at least three replicate HPF. In addition to absolute counts per high-power field, the proportion of $CD31^+$ endothelial cells positive for each marker was calculated within each field. Cell counts were

performed manually in Adobe Photoshop 2024 as in prior work from our group (6-9). A cell was considered positive for a marker when the following criteria were met: (i) fluorescent signal encircled a DAPI-positive nucleus; (ii) ≥50% of the nuclear perimeter was surrounded by signal; (iii) intensity was above local background. Double-positive cells required spatial overlap of both markers around the same DAPI-positive nucleus. All images were quantified by investigators blinded to diagnosis and sample identity. For each staining panel, a second independent blinded investigator quantified a subset of images to assess reproducibility. Inter-observer agreement was high, and discrepancies were resolved by joint review using the original scoring criteria. Image quantification visualization was performed in GraphPad Prism v10.

Endothelial cells were identified by expression of CD31. pMLKL signal was evaluated within a predefined cortical zone (0.5–1.5 μm immediately inside the CD31-defined endothelial border) corresponding to the cytoplasmic region where membrane-associated pMLKL accumulates during necroptosis (10). A cell was scored positive when at least two of the following features were present: rim-localized enrichment along the membrane, cortical puncta or short ribbons, intensity above local background, and nuclear sparing. Signal intensity above local background was required in all cases, and cells lacking this feature were not scored as positive. Cells without cortical enrichment, predominantly nuclear signal, or artifact were scored negative. Apoptotic endothelial cells were identified by co-expression of CD31 and cleaved caspase-3 or cleaved PARP. T_{RM} were defined as $CD3^+CD103^+$ or $CD3^+CD69^+$ cells with a $DAPI^+$ nucleus. Cytotoxic T cells were defined as $CD3^+CD8^+$. Only extraluminal T cells were included in all spatial analyses; intraluminal T cells were excluded. T_{RM} or cytotoxic T cells were considered spatially associated with a $pMLKL^+$ endothelial cell when located within approximately two cell

diameters (10-15 μm) of the CD31-defined vessel wall in the same optical plane. Endothelial apoptosis ($CD31^+$Cleaved-Caspase-3^+) or necroptosis ($CD31^+pMLKL^+$) was counted as T cell-associated when an affected endothelial cell had at least one nearby T cell: any $CD3^+$ T cell for apoptosis, or a $CD8^+$ or T_{RM} ($CD3^+CD103^+$ or $CD3^+CD69^+$) for necroptosis. Each such endothelial cell was counted as one T cell-associated dying endothelial cell event.

Statistical Analyses

Differentially expressed genes (DEGs) between adult morphea and healthy skin were identified in Cellenics, which uses a pseudobulk limma-voom workflow. Briefly, this workflow uses the voom pipeline from the limma package to compare gene-level expression between groups after aggregating single-cell counts by sample for each cluster. For endothelial analyses, lymphatic ECs and pericytes were excluded prior to DEG identification to restrict comparisons to vascular ECs. Genes were considered differentially expressed when comparison between groups yielded an adjusted P value < 0.05 and an absolute log2 fold change ≥ 1. The resulting DEG list was used as input for Ingenuity Pathway Analysis (IPA).

Ingenuity Pathway Analysis (IPA) (Qiagen) identified upstream regulators and canonical pathways in adult morphea vascular endothelial cells versus healthy controls. Core Analysis was performed with the full set of genes tested in this contrast as the reference background. Enrichment of IPA-curated canonical pathways was assessed by Fisher's exact test, with multiple-testing correction using the Benjamini-Hochberg false discovery rate (FDR) method. Upstream regulator analysis inferred transcriptional drivers of differential expression between morphea and healthy skin. Activation or inhibition status was assigned based on the IPA

activation Z-score, which integrates both the direction and magnitude of gene expression changes. Canonical pathways and upstream regulators were considered significantly dysregulated if $|Z| \geq 2$ and adjusted $P < 0.05$.

For box plots comparing T cell counts, frequencies, and endothelial subset proportions between groups, statistical significance was assessed using two-tailed unpaired t tests. For multiplex immunostaining analyses, significance was determined by two-tailed nested t tests. All statistical analyses were performed in GraphPad Prism v10, and differences were considered significant at $P < 0.05$.

Study Approval

All tissues were collected and analyzed under Institutional Review Board approval (Mass General Brigham Human Research Committee, protocol 2021P001443). De-identified healthy skin samples discarded from cosmetic procedures were obtained through the Human Skin Disease Resource Center at BWH and Harvard Medical School.

Data Availability Statement

This study analyzed the adult subset of a previously published single-cell RNA sequencing dataset, which is publicly available as described in Rosen et al., *JCI Insight*, 2025 (1). Data related to these studies, including significant DEG and IPA results, are provided in in the Supporting Data.

Acknowledgments

We thank the Human Skin Disease Resource Center (HSDRC) of Brigham and Women's Hospital and Harvard Medical School, which provided skin biopsies from healthy adults. The HSDRC is supported in part by the National Institute of Arthritis and Musculoskeletal and Skin Diseases Resource-based Center Grant P30AR069625-09. We also thank the patients with morphea at the University of Texas Southwestern Medical Center and the University of Pittsburgh Medical Center who contributed biopsy samples to the single-cell dataset analyzed in this study.

Author Contributions Statement

Conceptualization: WC, RC, AL; Formal Analysis: WC, NS; Funding Acquisition: RC; Investigation: WC, NS, SJS, MVM, AM, MW, TH, QZ; Methodology: WC, NS, SHS, AP, KST, HJ; Project Administration: JT; Supervision: WC, RC, AL; Visualization: WC, NS; Writing – Original Draft Preparation: WC, AL, RC; Writing – Review and Editing: JT, MJM, RAV

References

1. Rosen AB, Sanyal A, Hutchins T, Werner G, Berkowitz JS, Tabib T, et al. Unique and shared transcriptomic signatures underlying localized scleroderma pathogenesis identified using interpretable machine learning. *JCI Insight.* 2025;10(7).
2. Easter QT, Fernandes Matuck B, Beldorati Stark G, Worth CL, Predeus AV, Fremin B, et al. Single-cell and spatially resolved interactomics of tooth-associated keratinocytes in periodontitis. *Nat Commun.* 2024;15(1):5016.

3. de Almeida GP, Lichtner P, Eckstein G, Brinkschmidt T, Chu CF, Sun S, et al. Human skin-resident host T cells can persist long term after allogeneic stem cell transplantation and maintain recirculation potential. *Sci Immunol.* 2022;7(67):eabe2634.

4. Hutchins T, Sanyal A, Esencan D, Lafyatis R, Jacobe H, and Torok KS. Characterization of Endothelial Cell Subclusters in Localized Scleroderma Skin with Single-Cell RNA Sequencing Identifies NOTCH Signaling Pathway. *Int J Mol Sci.* 2024;25(19).

5. Jin S, Guerrero-Juarez CF, Zhang L, Chang I, Ramos R, Kuan CH, et al. Inference and analysis of cell-cell communication using CellChat. *Nat Commun.* 2021;12(1):1088.

6. Win TS, Crisler WJ, Dyring-Andersen B, Lopdrup R, Teague JE, Zhan Q, et al. Immunoregulatory and lipid presentation pathways are upregulated in human face transplant rejection. *J Clin Invest.* 2021;131(8).

7. Crisler WJ, Rowley R, Oke O, Steuart SJ, Modi MM, Davis DL, et al. Eosinophilic Fasciitis and Morphea Share Gene Signatures of Inflammatory Cell Death, Self-DNA Recognition, and Enhanced JAK/STAT Signaling. *J Invest Dermatol.* 2025.

8. Kauke-Navarro M, Crisler WJ, Younis N, Khetani RS, Sadigh S, Teague JE, et al. B-cell infiltration distinguishes mucosal from skin patterns of rejection in facial vascularized composite allografts. *Am J Transplant.* 2025.

9. Crisler WJ, Win TS, Kauke-Navarro M, Zhan Q, Barrera V, Sui SH, et al. Sentinel flaps reflect key inflammatory aspects of human face transplant rejection. *Am J Transplant.* 2025.

10. Samson AL, Zhang Y, Geoghegan ND, Gavin XJ, Davies KA, Mlodzianoski MJ, et al. MLKL trafficking and accumulation at the plasma membrane control the kinetics and threshold for necroptosis. *Nat Commun.* 2020;11(1):3151.

Supplementary Figures and Legends

Supplemental Figure 1. Single-cell and pathway analysis of T cells and endothelial cells in morphea. (**A**) Representative clinical image and H&E section from morphea lesions used for multiplex immunostaining. H&E shows dense pink collagen characteristic of morphea. (**B-C**) Dot plots depict defining marker genes for (**B**) T cell and NK cell subsets and (**C**) endothelial and perivascular cell subsets identified across all scRNA-seq samples. (**D**) UMAPs depict morphea and healthy skin (HS) samples, with expression of vascular endothelial (*PECAM1*, *VWF*), lymphatic (*PROX1*), and pericyte (*ACTA2*) markers. (**E**) Ingenuity Pathway Analysis (IPA) of pooled vascular endothelial cell (VEC) clusters, excluding lymphatic ECs and pericytes, showing activation and inhibition of mitochondrial/redox metabolism, integrated stress response, and nucleolar/redox quality control pathways. (**F**) Predicted upstream stress regulators in morphea VECs identified by IPA, grouped by metabolic, redox/repair regulation, and translational stress categories. Bars represent activation Z- score; red squares show -log10(p value). IPA P values were derived from Fisher's exact test with Benjamini-Hochberg correction. (**G**) CellChat analysis of single-cell RNA-seq data showing significant TNF ligand-receptor interactions from T cells to other cell populations in morphea ($p < 0.01$). (**H**) Stacked bar graph depicts proportions of endothelial cell subsets in morphea and HS.

Supplemental Figure 2. T_{RM}-associated endothelial apoptosis and necroptosis in morphea. (**A**) Number and percentage of apoptotic endothelial cells (CD31+cleaved-caspase-3+) per 200× high-power field (HPF) in morphea versus healthy skin (HS). (**B**) Multiplex immunofluorescence showing apoptotic (cleaved PARP+) endothelial cells (CD31+) adjacent to CD3+ T cells in morphea, with quantification of CD31+cleaved PARP+ endothelial cells near CD3+ cells per 200× HPF. (**C**) Number and percentage of apoptotic endothelial cells (CD31+cleaved PARP+) per 200× HPF in morphea versus HS. (**D, F, H**) Number and percentage of necroptotic endothelial cells (CD31+pMLKL+) per 200× HPF in sections stained with the CD8 (**D**), CD103 (**F**), or CD69 (**H**) panels. (**E, G**) Number and percentage of CD3+ T cells co-expressing CD103 (**E**) or CD69 (**G**) per 200× HPF. (**I**) Representative morphea lesion showing necroptotic (pMLKL+RIPK3+) endothelial cells (CD31+) near CD3+ T cells, with quantification of necroptotic endothelial cells adjacent to T cells in morphea and HS. Scale bar = 50 μm. Inset: arrowhead indicates T cell; arrow indicates necroptotic endothelial cell. Bars represent individual donors; error bars indicate mean ± SEM from at least three measurements per donor. Statistical significance was determined using two-tailed nested t tests. (**J**) Schematic model of T_{RM}-driven endothelial injury in morphea showing TNF and cytotoxic effectors acting on metabolically stressed, TNF-sensitized endothelial cells that undergo apoptosis or necroptosis, with proposed downstream activation of innate immune cells and fibroblast-mediated fibrosis.

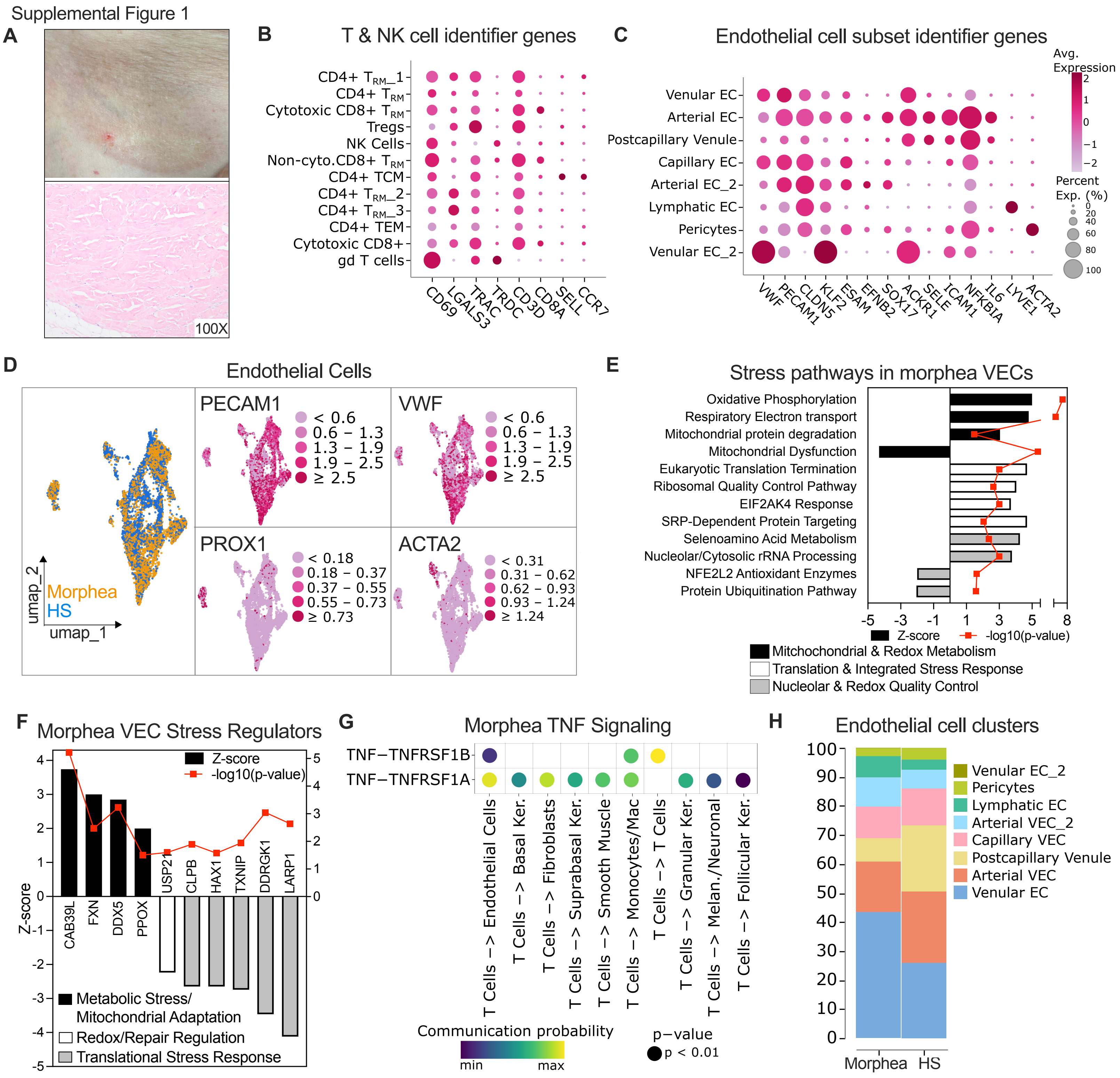

Supplemental Figure 1. Single-cell and pathway analysis of T cells and endothelial cells in morphea. (**A**) Representative clinical image and H&E section from morphea lesions used for multiplex immunostaining. H&E shows dense pink collagen characteristic of morphea. (**B-C**) Dot plots depict defining marker genes for (**B**) T cell and NK cell subsets and (**C**) endothelial and perivascular cell subsets identified across all scRNA-seq samples. (**D**) UMAPs depict morphea and healthy skin (HS) samples, with expression of vascular endothelial (*PECAM1*, *VWF*), lymphatic (*PROX1*), and pericyte (*ACTA2*) markers. (**E**) Ingenuity Pathway Analysis (IPA) of pooled vascular endothelial cell (VEC) clusters, excluding lymphatic ECs and pericytes, showing activation and inhibition of mitochondrial/redox metabolism, integrated stress response, and nucleolar/redox quality control pathways. (**F**) Predicted upstream stress regulators in morphea VECs identified by IPA, grouped by metabolic, redox/repair regulation, and translational stress categories. Bars represent activation Z-score; red squares show -log10(p value). IPA P values were derived from Fisher's exact test with Benjamini-Hochberg correction. (**G**) CellChat analysis of single-cell RNA-seq data showing significant TNF ligand-receptor interactions from T cells to other cell populations in morphea ($p < 0.01$). (**H**) Stacked bar graph depicts proportions of endothelial cell subsets in morphea and HS.

Supplemental Figure 2

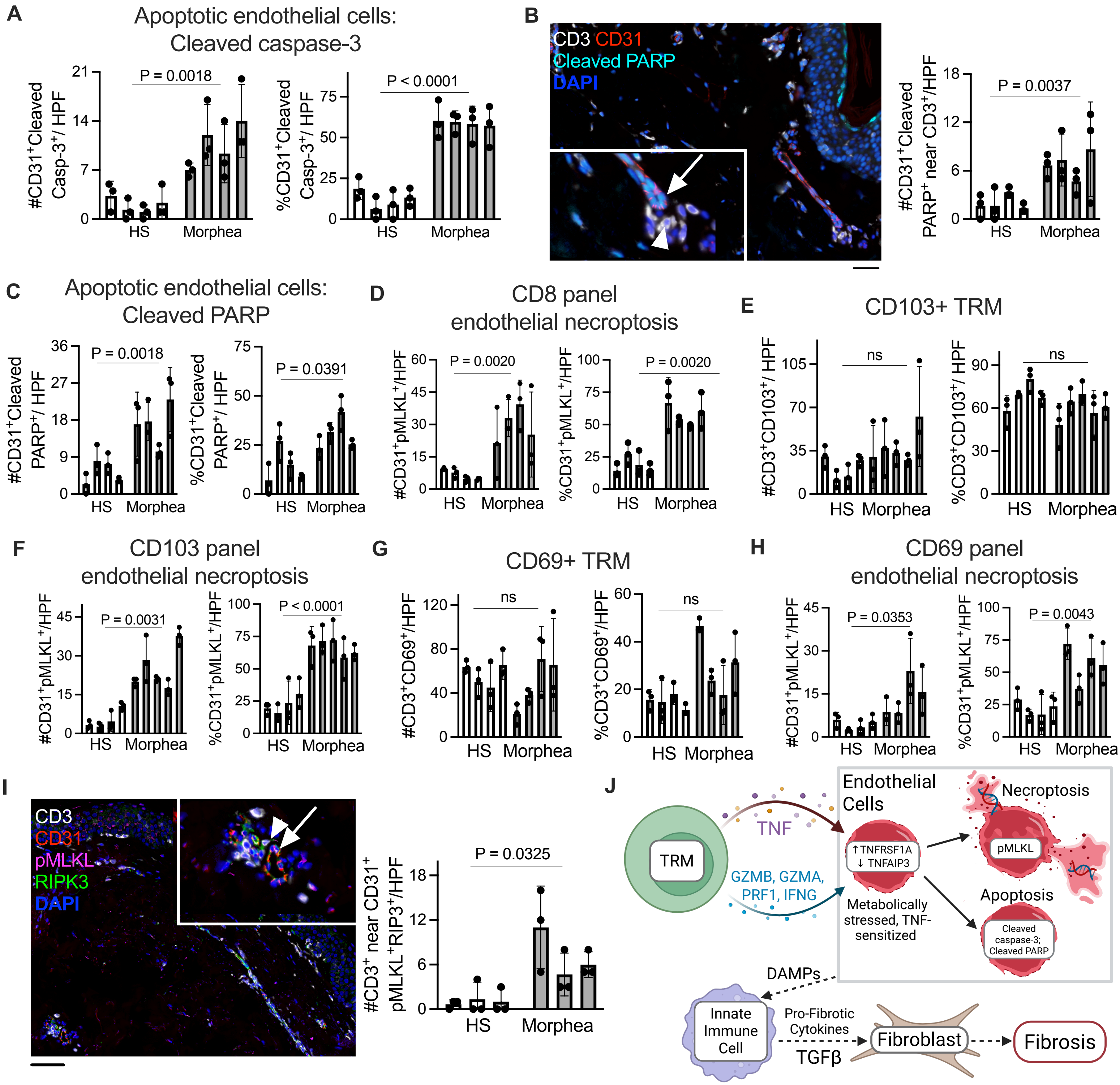

Supplemental Figure 2. TRM-associated endothelial apoptosis and necroptosis in morphea. (**A**) Number and percentage of apoptotic endothelial cells ($CD31^{+}$cleaved-caspase-3^{+}) per 200× high-power field (HPF) in morphea versus healthy skin (HS). (**B**) Multiplex immunofluorescence showing apoptotic (cleaved $PARP^{+}$) endothelial cells ($CD31^{+}$) adjacent to $CD3^{+}$ T cells in morphea, with quantification of $CD31^{+}$cleaved $PARP^{+}$ endothelial cells near $CD3^{+}$ cells per 200× HPF. (**C**) Number and percentage of apoptotic endothelial cells ($CD31^{+}$cleaved $PARP^{+}$) per 200× HPF in morphea versus HS. (**D, F, H**) Number and percentage of necroptotic endothelial cells ($CD31^{+}pMLKL^{+}$) per 200× HPF in sections stained with the CD8 (**D**), CD103 (**F**), or CD69 (**H**) panels. (**E, G**) Number and percentage of $CD3^{+}$ T cells co-expressing CD103 (**E**) or CD69 (**G**) per 200× HPF. (**I**) Representative morphea lesion showing necroptotic ($pMLKL^{+}RIPK3^{+}$) endothelial cells ($CD31^{+}$) near $CD3^{+}$ T cells, with quantification of necroptotic endothelial cells adjacent to T cells in morphea and HS. Scale bar = 50 μm. Inset: arrowhead indicates T cell; arrow indicates necroptotic endothelial cell. Bars represent individual donors; error bars indicate mean ± SEM from at least three measurements per donor. Statistical significance was determined using two-tailed nested t tests. (**J**) Schematic model of TRM-driven endothelial injury in morphea showing TNF and cytotoxic effectors acting on metabolically stressed, TNF-sensitized endothelial cells that undergo apoptosis or necroptosis, with proposed downstream activation of innate immune cells and fibroblast-mediated fibrosis.

Supplemental Table 1. Patient and Control Clinical Data. Abbreviations: LoSAI – Localized Scleroderma Activity Index; LoSDI – Localized Scleroderma Damage Index; H-Healthy; M-Morphea; F-Female; M-Male; A-Active; I-Inactive; G-Generalized, P-Plaque, L-Linear, Ind-Indeterminate

Study ID	Biopsy location	Age of onset	Disease duration at time of biopsy (years)	Age at biopsy	Sex	Race / Ethnicity	Activity Status	Sub-type	LoSAI	LoSDI
Control samples										
H1	Forearm			63	Male	Caucasian				
H2	Forearm			54	Male	Caucasian				
H3	Forearm			61	Male	Black				
H4	Forearm			66	F	Caucasian				
H5	Forearm			62	F	Caucasian				
H6	Forearm			63	Male	Caucasian				
H7	Forearm			64	Male	Caucasian				
H8	Forearm			48	F	Caucasian				
Patient samples										
M1	Abdomen	64	1	65	F	Caucasian	A	G	61	28
M2	Right arm	54	8	61	F	Caucasian	A	G	13	39
M3	Left abdomen			65	Male	Caucasian	A/I	G	14	38
M4	Lower back	34	13	43	Male	Caucasian	I	P	0	5
M5	Abdomen	24	13	37	Male	Caucasian	A	L	4	34
M6	Left abdomen	61	2.7	64	F	Caucasian	A	G	9	14
M7	Left abdomen	34	9	43	F	Hispanic	A	L	10	10
M8	Left flank	63	2	65	F	Caucasian	A	G	5	32
M9	Abdomen	61	10.8	71	F	Hispanic	A	L	5	13
M10	Abdomen	62	4	66	F	Caucasian	A	G	5	26
M11	Right thigh	47	0.3	47	F	Black	A	Ind	12	17
M12	Abdomen	27	1	27	F	Black	A	L	5	2
M13	Abdomen	57	8	65	Male	Caucasian	A	G	13	17

Supplemental Table 2. Clinical data for patient samples used for immunostaining. All patients in this cohort had active disease. Abbreviations: F-Female; CS-Corticosteroids, W-White, H-Hispanic, NH-Non-Hispanic, B-Black

Immuno-stain study ID	Biopsy location	Age at time of biopsy	Disease duration at time of biopsy	Sex	Race / ethnicity	Subtype	Therapy at time of biopsy	Figure(s) represented
1	Right abdomen	71	1.5 years	F	W, NH	Plaque	None	Fig 1K, Supplemental Fig 2B
2	Left hip	45	< 1 year	F	H	Plaque	None	Fig 1K, Supplemental Fig 2B
3	Left shin	68	< 1 year	F	W, NH	Plaque	None	Fig 1J, Fig 1K, Supplemental Fig 2B
4	Left upper back	54	< 1 year	Male	W, NH	Plaque	None	Fig 1I, Fig 1J, Fig 1K
5	Left chest	59	< 1 year	F	W, NH	Plaque	Topical CS	Fig 1I, Fig 1J, Supplemental Fig 2I
6	Left breast	25	5 years	F	W, NH	Plaque	None	Fig 1J, Fig 1K
7	Left chest	61	< 1 year	F	W, NH	Plaque	None	Fig 1K, Supplemental Fig 2B
8	Left inframammary	59	3 years	F	B, H	Generalized	Topical CS	Fig 1K
9	Right abdomen	62	< 1 year	F	W, NH	Plaque	None	Fig 1I, Supplemental Fig 2I
10	Right flank	36	>10 years	F	W, NH	Generalized	None	Fig 1I, Fig 1K, Supplemental Fig 2I
11	R shin	28	1 year	F	W, NH	Plaque	None	Fig 1K
